# Nanocarriers as treatment modalities for hypertension

**DOI:** 10.1080/10717544.2016.1255999

**Published:** 2017-02-06

**Authors:** Tausif Alam, Saba Khan, Bharti Gaba, Md. Faheem Haider, Sanjula Baboota, Javed Ali

**Affiliations:** Department of Pharmaceutics, Faculty of Pharmacy, Jamia Hamdard, Hamdard Nagar, New Delhi

**Keywords:** Antihypertensive, novel molecular targets for hypertension therapy, oral drug delivery constraints, nanotechnology, gene silencing

## Abstract

Hypertension, a worldwide epidemic at present, is not a disease in itself rather it is an important risk factor for serious cardiovascular disorders including myocardial infarction, stroke, heart failure, and peripheral artery disease. Though numerous drugs acting via different mechanism of action are available in the market as conventional formulations for the treatment of hypertension but they face substantial challenges regarding their bioavailability, dosing and associated adverse effects which greatly limit their therapeutic efficacies. Various studies have demonstrated that nanocarriers can significantly increase the drug bioavailability thereby reducing the frequency of dosing in addition to minimizing toxicity associated with high dose of the drug. The present review provides an insight into the challenges associated with the conventional antihypertensive formulations and need for oral nanoparticulate systems in order to overcome problems associated with conventional formulations. Hypertension has circadian pattern of blood pressure, therefore chronotherapeutics can play a decisive role for the treatment, and however, nanoparticulate system can play major role in hypertension management. Future prospective for particulate nanocarriers in drug delivery for hypertension includes chronotherapeutics and emerging technique like gene therapy which is also covered in the review.

## Introduction

Hypertension is a serious cardiovascular event which refers to rise in the arterial blood pressure. Due to raised blood pressure, heart has to work harder in order to pump adequate amount of blood to cope up with normal body functioning. If the same is not treated, it may lead to heart-related problems and may damage the organs like kidney, brain, and eyes. It is as such not a disease in itself but is a risk factor for major cardiovascular events like heart stroke, ischemic heart disease, myocardial infarction, and heart enlargement. According to WHO, Geneva, in 2008, hypertension resulted in 45% mortality rate because of ischemic heart disease and 51% mortality rate because of stroke. In 1980, 600 million people were suffering from hypertension while in 2008 this graph was raised to 1 billion raising a big concern for dealing with this condition effectively (WHO, 2013).

Several drugs in conventional dosage forms are available to treat hypertension but majority of the antihypertensives are poorly water soluble and therefore exhibits low bioavailability. These drugs are also substrate of Pgp and exhibit significant first-pass metabolism. The other challenges with these formulations are their short half-life and high dosing frequency. With the use of extended release systems, these dosing frequencies can be reduced but as far as enhancement of bioavailability is concerned nanoparticles are far better approach. The associated benefits with nanoparticle include their capability of circumventing first-pass metabolism, P-gp mediated efflux and achieving targeting because entrapped drug in carrier is directly taken into the systemic circulation.

Hindrance in the oral absorption of the drug includes extreme pH, poor intestinal permeability, and CYP 450-mediated enzymatic metabolism. Incorporation of the drug into nanoparticles can overcome these barriers (Pridgen et al., 2014). Proteins and peptides therapeutics including insulin glargine, etanercept, cyclosporine, desmopressin, and jellyfish collagen protein (possess antihypertensive activity) are poorly bioavailable due to their charged nature, high molecular weight, low lipophilicity, and degradation by protease and peptidase secreted in the GIT. Nanoparticles have been reported which increase the uptake of drug through different mechanism which includes transcellular absorption, paracellular transport by opening tight junction, P-gp inhibition, inhibition of gut wall metabolism by CYP450, and enhancement of lymphatic transport (Hauss, 2007). Nanoparticles of size 100 nm have been considered ideal for lymphatic transport of lipid nanoparticle (Ghosh & Roy, 2014). Solutol HS 15, poloxamer 188, polyethylene glycol, and Cremophor RH 40 are some surfactants used in formulating nanoparticles and show inhibition of P-gp efflux and CYP450 activity (Khan et al., 2015).

This review takes into account challenges associated with conventional antihypertensive formulations and role of oral nanoparticulate drug delivery system in overcoming such hurdles and enhancing the treatment of hypertension. The present review covers more recent and advanced technique for enhancing the efficacy of antihypertensive drugs. Most of the antihypertensive drug comes under BCS class 2 (low solubility and high permeability) which have low bioavailability as dissolution is the rate-limiting step. Drugs like amlodipine and isradipine apart from having low bioavailability are also light sensitive apart from being a BCS class 2 drugs. Delivery of such drug in protected form is required to prevent their photo-degradation. Both drugs were delivered by utilizing nanoemulsion as a drug delivery system. Their pharmacokinetic data revealed the stability and enhanced bioavailability (Jang D-J et al., 2006; Havanoor et al., 2014). Chronotherapeutics can deliver drugs at the time when symptoms occur like during night and early morning as in the case with hypertension. Chronotherapeutics in nanosize range can further be more effective and efficient in hypertension and has been discussed in liquid emulsion. Gene silencing is the recent technology where the use of small interfering RNA is done to silence those receptors which are involved in the increase of blood pressure.

Intravenous route is mostly used for the delivery of SiRNA in treating hypertension. Incorporation of SiRNA in delivery system is required to prevent their degradation by exonuclease activity present in blood (Nolte et al., 2011; McLendon et al., 2015). Oral delivery has been rarely studied for the delivery of SiRNA for the treatment of hypertension. Researches are available for the oral delivery of SiRNA for other diseases. Knipe et al. (2016) has developed microencapsulated nanogel for the oral delivery of SiRNA to treat inflammatory bowel disease by targeting TNF-α. In another study, galactose-modified trimethyl chitosan-cysteine conjugates with various galactose grafting densities were formulated to delivery vascular endothelial growth factor SiRNA orally for the treatment of hepatoma (Han et al., 2014).

## Antihypertensive drug therapy

The first drug which was developed for treating hypertension was pentaquine in 1946 but it showed several side effects with little therapeutic efficacy. Soon in early 1950s, ganglionic blocking agent “hexamethonium” was introduced which was efficacious but was not convenient to use. Veratrum was introduced which had short onset of action but was toxic. Hydralazine developed soon after seeing the side effects of ganglionic blockers and is seldom prescribed today. Reserpine, the most effective drug developed at that time, was also abandoned due to its side effects like depression and impotency. Breakthrough drugs like diuretics and β-blockers which are highly widely prescribed today are named as the modern era of antihypertensive which was started in 1960.

In 1990s, calcium channel blockers, angiotensin converting enzyme inhibitors, and angiotensin blockers were introduced which are now prescribed as the first-line therapy either alone or in combination.

**Figure 1.**
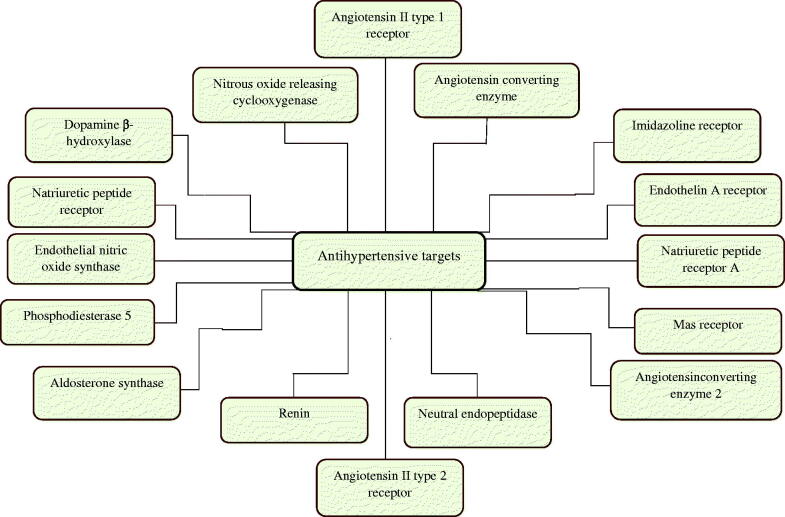
Novel molecular targets for antihypertensives.

Thorough understanding of renin angiotensinogen aldosterone system (RAAS) has led to the development of several antihypertensives. There is dramatic progress in the development of novel therapeutics, the target of which is also related to RAAS (Paulis & Unger, 2010). Figure 1 shows the novel targets which have opened the new possibility for the successful development of the drug for treating hypertension which are currently under preclinical and clinical stages of development. Various antihypertensive drugs including novel antihypertensive with their class, mechanism of action, and the development stage are described in Table 1.

**Table 1.** Some novel antihypertensives with their development phase and mechanism of action.

Mechanism	Drug	Development phase	Company	References
Aldosterone-receptor blocker	Eplerenone	Marketed	Pfizer, USA	ACE 2 modulator
Phosphodiesterase 5 inhibitor	TadalafilKD027	MarketedPhase II	Eli Lilly, USAKadmon Pharmaceuticals	ACE 2 modulator ; Adis Insight
Dopamine β-hydroxylase inhibitor	Etamicastat	Phase I	Bial, Portugal	McLendon et al. (2015)
ACE 2 modulator	APN01 (rhACE2)	Phase II	Apeiron-biologics	Morrell et al. (2013)
Aldosterone synthase inhibitor	ASI LCI699	Phase II	Novartis, Switzerland	Muller et al. (2000)
ACE inhibitor	Imidapril	Marketed	Mitsubishi Tanabe Pharma	Nolte et al. (2011)
AT1R blocker with PPAR-γ activity	Azilsartan (TAK-491)	Marketed	Takeda Pharmaceuticals, Japan	Novartis' new heart failure medicine LCZ696, now called Entresto(TM), approved by FDA to reduce risk of cardiovascular death and heart failure hospitalization [Online]
AT2R agonist	Compound 21	Phase I	Vicore, Sweden	Nunes et al. (2010)
Combined AT1R blocker and NEP inhibitor	LCZ696Daglutril	Phase IIIPhase II	Novartis, SwitzerlandSolvay, Belgium	O'Driscoll & Griffin (2008); Ohara-ch [Online]
Renin inhibitor	AliskirenVTP27999	MarketedPhase II	Novartis, Switzerland, and Speedel SwitzerlandVitae Pharmaceuticals, USA	ACE 2 modulator; Oparil & Schmieder (2015)
Endothelin A receptor antagonist	Macitentan (PAH)Ambrisentan (PAH)	MarketedMarketed	Actelion PharmaceuticalsGilead	Palatin Technologies, Inc [Online]; Paulis & Unger (2010)
Imidazoline-receptor blocker	Monoxidine	Marketed	Eli Lilly, USA	Paulis et al. (2015)
Natriuretic peptide receptor agonist	PL3994	Phase II	Palatin Technologies, USA	Pridgen et al. (2014)
Endothelila nitric oxide synthase coupler	Cicletanine	Marketed	Gilead Sciences, Inc	Antal et al. (2015); Ranpise et al. (2014)
NO-releasing COX inhibitor	Naproxcinod	Phase III	NicOx, France	Selvamuthukumar & Velmurugan (2012).
Mas GPCR receptor	CGEN-856	Preclinical	BioLineRx	Shafiq et al. (2007)

PAH: pulmonary arterial hypertension, ACE: angiotensin-converting enzyme, AT1R: angiotensin II type 1 receptor, AT2R: angiotensin II type 2 receptor, PPAR-γ: peroxisome proliferator-activated receptor gamma.

### Renin inhibitor

Renin is released from kidney and acts as a first step for the RAAS cascade. So, it acts as a target for antihypertensive therapy. In 2007, aliskiren the first-marketed renin inhibitor was introduced into the market. VTP27999 is a new molecule which is under Phase II clinical trial (Paulis et al., 2015).

### Angiotensin II type 2 receptor agonist

AT2R has action opposite to that of AT1R. It opposes the AT1R-mediated vasoconstrictor action of angiotensin II. AT2R shows vasodilatory action which is formed of bradykinin, nitric oxide, and cGMP. AT2R also mediates natriuresis (Carey & Padia, 2008). Compound 21 is AT2R agonist which is under clinical trial for its antihypertensive action. It acts on the sodium/hydrogen exchanger 3 (NHE 3) and the Na^+^/K^+^-ATPase in the proximal tubules, thus showing natriuresis (Paulis et al., 2015).

### Phosphodiesterase 5 (PDE-5) inhibitor

PDE-5 causes degradation of cyclic GMP which is the intermediate step in vasodilatory action. PDE-5 inhibits cGMP degradation thereby causing vasodilatation (Nunes et al., 2010). Tadalafil is recently approved PDE-5 inhibitor showing vasodilatory effect; KD027 is the another PDE-5 inhibitor which is under phase II clinical trial of study (Paulis et al., 2015).

### Natriuretic peptide receptor a (NPRA) agonist

Atrial and brain natriuretic peptides cause vasodilatory effect through cGMP by acting on NPRA. So, NPRA agonist like PL3994 which is under phase II trial of study causes increase in cGMP level leading to decrease in the blood pressure and induction of natriuresis (Paulis et al., 2015).

### Mas receptor modulator

Mas receptor like AT2 receptor causes release of nitrous oxide. Blockage of either Mas receptor or AT2 causes the blockage of other receptor due to their hetero-dimerization. Natural ligand for AT2 receptor is angiotensinogen, while for Mas receptor, it is Ang (1–7). Ang (1–7) has low bioavailability which can be enhanced by complexing it with hydroxyl-propyl β-cyclodextrin. It is still under preclinical trial of study (Paulis et al., 2015).

### Angiotensin converting enzyme 2 (ACE2) modulator

ACE2 causes metabolism of angiotensin I and angiotensin II which are the key peptides for RAAS. Ang (1–7) is the metabolic product of ACE2 as described above. Angiotensin-converting enzyme 2 (ACE2) modulator like APN01 (rhACE2) which are under Phase II clinical trial are showing promising results in controlling rise in blood pressure through this mechanism of action (Paulis et al., 2015).

### Endothelin a receptor (ET_A_) antagonist

Endothelin specially ET1 binds with endothelin receptor ET_A_ and/or ET_B_ expressed on cell membrane and produces effects like systemic and pulmonary vasoconstriction apart from oxidative damage, atherosclerosis, fibrinogenesis, and salt and water retention. Macitentan and Ambrisentan are recently approved Endothelin A receptor (ET_A_) antagonist for pulmonary hypertension which shows its effect by preventing the binding of ET1 to both ET_A_ and ET_B_ (Iglarz et al., 2008; Paulis et al., 2015).

### Combined AT1R blocker and NEP inhibitor

Neutral endopeptidase also called as neprilysin, vasopeptidase, or enkephalinase degrades various peptide hormones into inactive fragments. These hormones are angiotensin I, II, and endothelin (vasoconstrictor). NEP also degrades vasodilators like natriuretic peptides and kinins. NEP inhibition increases the bioavailability of Natriuretic peptides which contribute to lowering of blood pressure but at the same time level of vasoconstrictor also gets increased. So, the strategy is to design a drug with AT1R or ACE blocking activity. Daglutril and LCZ696 which are under phase II and III trial are NEP inhibitor with AT1R blocking activity (McMurray, 2015; Paulis et al., 2015).

### Imidazoline-receptor blocker

Imidazoline receptors are of 3 types: I1, I2, and I3. I1 imidazoline receptor mediates the sympathoinhibitory actions to lower blood pressure. I1 receptors present in the rostral ventrolateral medulla oblongata (RVLM) are stimulated by clonidine, a first-generation imidazolines. But this drug also show binding capacity for alpha 2 receptor thus producing side effects. Second-generation centrally acting antihypertensives like monoxidine are more selective for I1 than for alpha 2 receptor thus comparative less toxic than first-generation antihypertensives (Ernsberger et al., 1994; Head & Mayorov, 2006).

### Endothelilal nitric oxide synthase (eNOS) coupler

Patients with pulmonary artery hypertension have low levels of a substance called nitric oxide (NO) which maintains the normal tone of blood vessel. Endothelial nitric oxide synthase (eNOS) is the enzyme required for the production of NO. When eNOS is uncoupled (not dimerized), the production of NO is decreased apart from formation of reactive oxygen species (ROS). Both of these activities result in enhanced vasoconstriction. Cicletanine which is a thiazide like diuretic also acts as eNOS coupler which makes eNOS active thus increasing NO production and decreasing ROS formation (Labato, 2008).

### NO-releasing cyclooxygenase (COX) inhibitor

Naproxcinod is non-steroidal antiinflammatory drug which acts as cyclooxygenase inhibiting nitric oxide donor (CINOD). NO so produced has vasodilatory effect which developed the interest of the researcher to test it for blood pressure lowering effect and is currently under phase III trial of study (Townsend et al., 2011).

### Aldosterone synthase inhibitor

Mineralocorticoid receptor antagonists are not effective in reducing non-genomic effect of aldosterone. So, aldosterone synthase has drawn the attention toward mediating the blood pressure. Aldosterone synthase is cytochrome P450 enzyme involved in the biosynthesis of aldosterone. Inhibitor of this enzyme ASI LCI699 (in phase II trial) results in disruption of RAAS to keep rise in blood pressure under check (Niaz et al., 2016).

### Dopamine β-hydroxylase (DβH) inhibitor

DβH is the enzyme which hydrolyzes the neurotransmitter dopamine into nor-adrenaline in sympathetic nervous system which acts on α-receptor to produce vasoconstriction. DβH inhibitor, etamicastat, causes vasodilation, natriuresis, and diuresis (Nunes et al., 2010).

## Constraints with oral delivery of antihypertensive

Generally solubility and permeability are the prerequisite for the oral absorption of the drug. Certain antihypertensives like deltiazem, nicardipine, and nifedipine are the candidate for the P-glycoprotein (P-gp)-mediated efflux transporter present in the intestinal wall apart from Cytochrome P450-mediated enzymatic metabolism (O'Driscoll & Griffin, 2008; Basalious et al., 2010; Hetal et al., 2010; El-Kattan & Varma, 2012; Voruganti et al., 2010; Zisaki et al., 2015). Drugs which are under BCS class 2 show variable absorption pattern and low bioavailability. Most of the antihypertensive comes under BCS class 2, some of which are mentioned in Table 2 which represents that drug metabolism, solubility, permeability (log P), and P-gp are the critical parameters which determine the bioavailability of the antihypertensive.

**Table 2.** Physicochemical and metabolic profile of antihypertensives showing poor oral bioavailability.

Class	Drug	Metabolism	Solubility	Log P	Bioavailability
Calcium channel blocker	Nisoldipine	Extensive gut wall metabolism, CYP3A4 substrate	5.7 μg/ml	3.1	<5%
	Nitrendipine	Extensive hepatic first-pass metabolism by CYP3A4	2 μg/ml	3.59	10–20%
	Lacidipine	Completely metabolized in liver by CYP3A4	0.84 μg/ml	5	10%
	Verapamil	Extensively metabolized by CYP2C8, CYP2C18, and CYP2C9	7 mg/ml	3.8	10–20%
	Nifedipine	Hepatic metabolism by CYP3A4	20 μg/ml	2.20	45–56
	Amlodipine	Metabolized by CYP3A4	75.3 μg/ml	2.22	64%
	Felodipine	Inclusively metabolized by CYP3A4	7.15 μg/ml	4.36	15%
AT1 blocker	Olmesartan	Not metabolized by cytochrome P450 but is metabolized by liver esterase	7.75 μg/ml	5.5	26%
	Valsartan	CYP2C9	< 0.1 mg/ml	5.8	<25%
Beta blocker	Carvedilol	CYP1A2, CYP3A4, CYP1A1 CYP2D6, CYP2E1, CYP2C9	0.583 μg/ml	4.1	20%
Renin Inhibitor	Aliskiren	CYP3A4-mediated hepatic metabolism	122 mg/ml as hemifumarate salt	2.45	2.5%

Nanoparticles seem to be the better approach to remove the constraints related with oral delivery of antihypertensive. Different nanoparticulate systems like polymeric nanoparticles and lipid-based nanoparticles (nanoemulsion, SLN, NLC, lipotomes) have been studied to overcome limitations associated with the oral delivery of antihypertensive. Table 3 shows the advantages of using nanoparticle over conventional therapy.

**Table 3.** Novel delivery system of antihypertensives and their positive outcome.

Type of delivery system	Therapeutic system	Excipients used	*In-vivo* study model	Comments
Polymeric nanoparticle	Ramipril	lecithin/chitosan	Male Wistar rats	1.6-fold decrease in systolic blood pressure
	Nifedipine	PCLPLAGAEudragit RL/RS	Male adult SHR	Initial fall in systolic blood pressure was rapid for PEG solution followed by with PCL NP and PLAGA NP.Blood pressure was within normal range after 10 h of dosing with all three NPs while PEG solution failed to achieve such sustained effect.
	Felodipine	PLGA, Pluronic F-68	Male Wistar rats	Systolic blood pressure normalized and elevated ST segment of ECG became normal upto a period of 3 days as compared to drug suspension.
	Lercanidipine	HPMC, TPGS	Male Sprague–Dawley rats	2.47 increase in oral bioavailability than raw drug without TPGS
	Aliskiren	Magnetite, poly (D, L-lactide), Pluronic F-68	Male spontaneouslyhypertensive rats	Significant decrease in mean systolic blood pressure by aliskiren nanoparticle as compared to aliskiren suspension and placebo
Solid Lipid nanoparticle	Nisoldipine	Trimyristin (TM; Dynasan-114; glyceryl trimyristate), egg lecithin, Poloxamer-188	Male Wistar rats	2.17 times increase in oral bioavailability, significant reduction in systolic blood pressure for a period of 36 h
	Candesartan Cilexetil	GMS, soy lecithin, Tween 80	Male Sprague–Dawley rats	12 times increase in oral bioavailability
	Isradipine	Trimyristin or GMS, poloxamer 188	Wistar rats	Significant decrease in the systolic blood pressure with SLN formulation using two different lipids
Nanostructured Lipid Carrier	Lacidipine	GMS, Linoleic acid and poloxamer 407	Wistar male albino rats	3.9 times enhancement in the relative bioavailability
	Lercanidipine	Labrafil 2130M, GMS, linseed oil and Tween 80	Male Sprague–Dawley rats	24 h control on the blood pressure by NLC as compared to plain drug suspension
Nanoemulsion	Ramipril	Sefsol 218, Tween 80, carbitol	Wistar male albino rats	229.62% increase in relative bioavailability of ramipril nanoemulsion as compared to ramiprol marketed capsule and 539.49% increase in bioavailability of formulation as compared to drug suspension.
	Amlodipine	DE (Labrafilm 1944 CS and Dextrin)	Male Sprague–Dawley rats	In vitro release studied showed higher release of amlodipine from DE than powdered drug. 2.6 to 2.9 times increase in C_max_ and AUC (0–24h) from DE than powder. Marked reduction in photodegradation of drug in DE than powdered drug (5.6% versus 66.9%)
	Olmesartan Medoxomil (Beg et al., 2015)	SNEDDS (SNEOF and CSNEOF)	Unisex Wistar rats	After 0.5 h of dosing, significant reduction in arterial blood pressure (180 to 189 mm Hg) was seen with SNEOF (141 ± 1.36), CSNEOF (136 ± 1.45), and marketed formulation (138 ± 1.98). After 48 h of study, rats were found normotensive (BP < 130 mm Hg) with SNEOF and CSNEOF
	Valsartan	S-SNEDDS (Capmul MCM, Labrasol, Tween 20)	Male Wistar rats	3–3.5 time increase in the rate of dissolution, significant reduction in the mean systolic blood pressure after 0.5 h and 2 h of dosing of S-SNEDDS as compared to valsartan suspension showing faster onset of action of S-SNEDDS thus showing it to have the potential of the bioavailability enhancement of valsartan
	Lacidipine	S-SNEDDS (Labrafil and capmul as oil, Cremophor and Tween 80 as surfactant and transcutol as co-surfactant)	Male Wistar rats	Rate of dissolution increased significantly
	Carvedilol	S-SNEDDS (Capmul MCM, Nikkol HCO 50) L-SNEDDS (Cremophor EL, Transcutol HP)	———	2.34 and 1.85 times enhancement in C_max_ and AUC, respectively of S-SNEDDS, thus showing increase in the bioavailability.
Lipotomes	Lacidipine	Cetyl alcohol and Tween 80	Adult male human volunteer	540.11% increase in relative bioavailability of enteric-coated capsule of lipotome as compared to Motens tablet

PCL: poly-e-caprolactone, PLAGA: polylactic and glycolic acid, PEG: Polyethylene glycol, NP: Nanoparticle, SHR: systolic hypertensive rat, DE: Dry emulsion, SNEDDS: Self-Nanoemulsifying drug delivery system, S-SNEDDS: solid self nanoemulsifying drug delivery system, HPMC: Hydroxypropyl methyl cellulose, TPGS: D-α-tocopheryl polyethylene glycol1000 succinate, SNEOF: Self nanoemulsifying oily formulation, CSNEOF: Cationic Self nanoemulsifying oily formulation, GMS: Glyceryl monostearate.

Polymeric nanoparticles offer the advantage of protecting pH-sensitive antihypertensives like nisoldipine eudragit nanoparticle and chitosan-based nanoparticle which degrades at intestinal pH as discussed under Section Polymeric nanoparticles. Magnetic nanoparticle can be targeted to a place where drug release is required through externally applied magnetic field. This property can reduce the dose of the drug and decrease the adverse effect (Akbarzadeh et al., 2012). Aliskiren possess low oral bioavailability of 2.6% due to its poor absorption as the permeability of the drug through GIT is low. Poly(D,L-lactide) (PLA) magnetic nanoparticle of aliskiren when administered intravenously showed better AUC and control on blood pressure (Antal et al., 2015). However, there is requirement of external magnetic field. Olmesartan medoxomil, valsartan, lacidipine, and carvedilol show poor bioavailability due to low aqueous solubility and enzymatic degradation. Self-nanoemulsifying drug delivery system, a type of nanoemulsion, shows positive outcome when formulated using these drugs (Table 3). But such kind of emulsion is associated with some inherent limitations such as storage stability due to creaming, Ostwald ripening, and expensive manufacturing process due to requirement of special instruments (Sharma et al., 2010). Solid lipid nanoparticles of Candesartan, nisoldipine, and isradipine were developed to increase oral bioavailability of these drugs. SLN consists of solid lipid surrounded by surfactants and has the advantages of both liquid emulsion and polymeric nanoparticle. They are made of biological lipids, so they are biocompatible and are biodegradable. Unlike polymeric nanoparticle, they are produced without utilizing organic solvents (Shah et al., 2014a).

Nanoemulsion has higher solubilization capacity so it can entrap higher amount of drug. It is thermodynamically stable, has higher shelf-life on storage, and is rapid acting. Target delivery can also be achieved with this delivery system. Low oral bioavailability of amlodipine due to low aqueous solubility and low permeability decreases the concentration of drug into its target site i.e. heart (Chhabra et al., 2011). Nanoemulsion increases the aqueous solubility and thus bioavailability of the drug which is revealed through the pharmacokinetic data obtained. There is also some problems associated with SLN like low drug load and expulsion of drug with time from the lipid matrix due to crystallization of solid lipid. So, a second generation of lipid nanoparticulate system known as nanostructured lipid carrier was developed which overcomes limitations associated with the SLN due to involvement of liquid lipid in the solid lipid shell. This liquid lipid solubilizes more amount of the drug and also does not crystallizes with time so greater entrapment efficiency, loading capacity, and stability is achieved (Beloqui et al., 2016). Lacidipine and lercanidipine NLC are low water soluble and show variable absorption pattern. They are also prone to systemic first-pass metabolism. Their inherent disadvantages were overcome by entrapping them in NLC. Result of this formulation is summarized in Table 3.

## Nanotechnology-based oral delivery of antihypertensive

### Rationale for using nanocarriers

Oral route is the most preferred route for the administration of the drugs. But the delivery of drug exhibiting low aqueous solubility and/or permeability (BCS class II or IV) is very challenging as bioavailability of these drugs is very low and pH of the GIT also varies from acidic in stomach to basic in the intestine. The pH of the GIT varies from 1 in stomach to 8 in the intestine (Koziolek et al., 2015). This wide difference in the pH can severely hamper the pharmacological activity of the drug by oxidation, deamidation, or hydrolysis of protein drugs. Oral bioavailability of drugs like candesartan cilexetil is affected as they undergo chemical degradation at acidic pH. Enzymes like liver esterase and cytochrome P450 cause significant degradation of antihypertensives as shown in Table 2. Protease degrades 94–98% of orally administered protein. Intestinal mucosa is the other barrier which hinders drug permeation. Mucosal barrier consists of extrinsic barrier (microenvironment near the vicinity of mucus layer) and intrinsic barrier (epithelial cell monolayer). Intrinsic barrier is due to the presence of tight junction between adjacent cells. Different mechanism by which any molecule can cross this barrier includes transcellular, paracellular, and transcytosis. Transcytosis being active transport pathway restricts large sized and charged molecules. When the mucosal barrier is permeated, molecules have to cross lamina propria where blood capillaries lie and molecule can get entry into the blood stream (Turner, 2009; Pridgen et al., 2014). Strategy to overcome intestinal barrier was to prepare mucoadhesive formulation which increases the contact time of the formulation with mucus thereby increasing drug concentration at the site of absorption. Many mucoadhesives have the property of acting as permeation enhancer which can open tight junction and paracellular transport becomes possible. Another way to enhance GI permeability is transport through M cells. M cells have less quantity of protease enzyme and lacks mucus secretion. Lipophilic molecules have improved M cell transport (Pridgen et al., 2014).

Lipid nanoparticles like SLN and NLC are transferred through intestinal barrier by clathrin-mediated transport. SLN is also transcytosed by caveolae-mediated endocytosis while NLC is transported by paracellular transport through tight junctions (Neves et al., 2016). Different nanoparticulate systems have been investigated to circumvent first-pass metabolism through lymphatic transport and includes nanoemulsion, liposome, SLN, and NLC. Size range of 100–500 nm has been proposed to be ideal in the lymphatic uptake but rate of absorption is faster when size is below 100 nm. Negatively charged nanoparticles show higher lymphatic uptake than positively charged and neutral nanoparticles. Lipophilicity acts as an add-on for lymphatic uptake of drug. NLC of hydrophilic drugs acts as a better approach for enhancing the uptake of such drugs (Ghosh & Roy, 2014). Furthermore, efflux transporter like P-glycoprotein present on the intestinal wall causes efflux of several antihypertensive leading to poor oral bioavailability (Desai et al., 2012). Drug encapsulated in nanoparticle can avoid all these constraint and sustained action can also be achieved leading to dose reduction and frequency of dosing (Niaz et al., 2016).

### Currently used nanocarriers for the antihypertensive drug delivery

**Figure 2.**
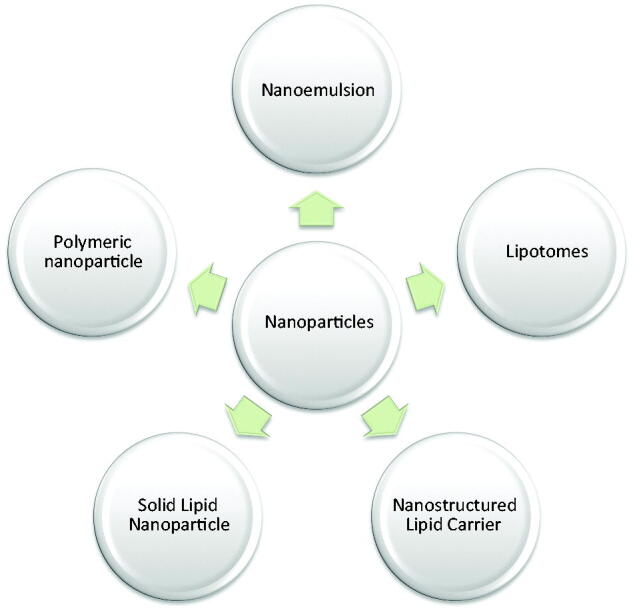
Diagram for currently used nanoparticles utilized in the treatment of hypertension.

Figure 2 gives an overview of currently used nanoparticles for the treatment of hypertension. Different nanocarriers which have been used for oral delivery of antihypertensive fall into different categories as mentioned in Table 3. The materials used in the preparation of nanoparticles must be nontoxic and biodegradable. Application of different nanoparticle for oral delivery has been discussed in the upcoming sections.

#### Polymeric nanoparticles

Polymer-based nanoparticles which have been extensively studied for oral antihypertensive include polylactide-co-glycolide (PLGA), poly-e caprolactone (PCL), Eudragit RL/RS, hydroxy propyl methyl cellulose (HPMC), and chitosan. Drug release from these nanoparticles is influenced by the method of preparation, particle size, surfactants, molecular weight of polymer, and polymer architecture (Desai, 2012).

Some drugs are pH sensitive like artemether, erythromycin, and candesartan cilexetil and to prevent acidic degradation they need to be targeted in intestine or colon-specific region. The pH-sensitive polymers can target drug at specific area of GIT. For drugs which are degraded in acidic environment, methacrylic acid, copolymers like EudragitS100/L100, can be used to target the colon while drugs which are susceptible to degradation at lower part of GIT Eudragit L100-55 can be used for the drug delivery at controlled rate (Desai, 2012). Application of these polymers is wide which include tissue targeting, delivery of biotherapeutics, and enhancing drug solubility. Rate of drug release from these polymers is fast. Drug release at the site of absorption creates the concentration gradient which helps in permeation of drug from the site of absorption thus causing increase in the drug bioavailability. Nepolean et al. prepared nisoldipine Eudragit S100 nanoparticle. It was studied that the release of drug from the polymer was pH responsive and was evident to occur at the pH of the colon. There was avoidance of drug metabolism mediated by cytochrome P450 in the liver and gut wall. Thus it was concluded that the formulation has the capability to enhance oral bioavailability of the drug (Nepolean et al., 2012). Kim et al. (1997) prepared three different NPs of PLGA, PCL, and eudragit for delivering nifedipine. Initial fall in systolic blood pressure was rapid for PEG solution (193 ± 3 mm of Hg to 102 ± 2 mm of Hg) compared to Eudragit nanoparticle (189 ± 2 mm of Hg to 156 ± 2 mm of Hg) while significant reduction in blood pressure was seen with PCL NP (189 ± 2 mm of Hg to 124 ± 2 mm of Hg) and PLGA NP (113 mm of Hg ± 2 mm of Hg). After 10 h, blood pressure with PEG solution of nifedipine was returned to normal while there was still significantly reduced blood pressure with all three NPs.

PLGA [Poly(lactic-co-glycolic acid)] are made up of lactic acid and glycolic acid monomer which are endogenous and are degraded easily, so the toxicity associated with these NPs is minimal. PLGA is US FDA and EMA approved. They are available in different form depending upon the ratio of the monomer. They can entrap both hydrophilic and hydrophobic drug and can provide sustained release profile from days to years depending upon the ratio of the monomer. They can also be used to target specific tissue or organ after modifying their surface (Danhier et al., 2012). Shah et al. prepared PLGA NPs of felodipine. Systolic blood pressure normalized and elevated ST segment of ECG came under control for a period of 3 days when compared with drug suspension (Shah et al., 2014b).

In another study, aliskiren magnetic nanoparticles were prepared using magnetite (Fe_3_O_4_) as magnetic material and poly(D,L-lactide) as polymer. Decrease in systolic blood pressure to 153.8 ± 3.9 mm of Hg as compared to placebo and aliskiren suspension having mean systolic blood pressure of 203.4 ± 4.3 mm of Hg and 178.7 ± 1.8 mm of Hg, respectively, revealed the success of the study in treatment of hypertension (Antal et al., 2015).

##### Chitosan nanoparticle

Chitosan is natural biodegradable, biocompatible, and nontoxic to human body. Chitosan is bioadhesive linear polysaccharide which is used as sustained release and site-specific delivery system for many drugs, including antihypertensive. Chitosan nanoparticles have enhanced the oral bioavailability of antihypertensive by preventing first-pass metabolism and degradation at acidic pH at upper GIT as chitosan are degraded by colonic microbes where pH is basic (Niaz et al., 2016). Chadha et al. (2013) prepared ramipril-*β*-Cyclodextrin complexed nanoparticles of lecithin/chitosan. *In vivo* result showed 1.6 times decrease in systolic blood pressure of deoxycorticosterone acetate salt induced hypertensive rats. Chitosan nanoparticles emerged as a solution for oral administration of antihypertensive which are poorly soluble.

##### Hydroxypropyl methyl cellulose (HPMC) nanoparticle

HPMC is nonionic water-soluble derivative of cellulose ether used for preparing controlled release dosage form. They are available in different viscosity depending upon the concentration of methoxy and hydroxypropoxyl group (16.5–30% of methoxy and 4.0–32.0% of hydroxypropoxy groups) (Ishikawa, 2000). Swelling and erosion of HPMC depends upon the pH and ionic strength of release media (Zabihi et al., 2015). HPMC has been used to deliver several antihypertensives showing poor bioavailability as tablet dosage form. This property of the HPMC was utilized in conjunction with TPGS as surfactant by Ha et al. to enhance the dissolution and bioavailability of lercanidipine which was estimated to be 2.47 times to that of pure lercanidipine.

#### Lipid-based nanoparticles

Several antihypertensives have been prepared using lipid-based delivery system as mentioned in Table 3. Lipid-based nanoparticles are ideal candidate for drug delivery of antihypertensive showing low solubility and high permeability. Lipid-based excipients can entrap greater amount of lipophilic drug than hydrophilic drug. Lipid nanoparticles (LNs) entrapped drug which are poorly soluble; the dissolution step is not needed as the drug is generally solubilized in lipid excipients (Kuentz, 2012). This solubilization is generally maintained throughout the gastrointestinal passage. The excipients used in preparing LNs include surfactants and co-surfactants apart from lipid which can promote permeability across intestinal wall. The mechanism underlying the enhancement of drug absorption includes increase in membrane fluidity, opening of tight junction, inhibition of P-glycoprotein efflux transporter, alteration of intestinal metabolism mediated by cytochrome P450, and lymphatic uptake thus by-passing hepatic first-pass metabolism. Various lipid-based nanoparticles which have been used in loading antihypertensive drugs are discussed below.

##### Liquid emulsion

Liquid emulsion (LE) includes self-microemulsifying drug delivery system (SMEDDS), self-nanoemulsifying drug delivery system (SNEDDS), microemulsion, and nanoemulsion. Nanoemulsion and self-emulsifying systems have been discussed in separate subheadings.

##### Nanoemulsion

Nanoemulsion is thermodynamically stable drug delivery system which can solubilize higher amount of drug. It is rapid acting, has higher shelf-life, and can be targeted. This system can achieve high oral bioavailability (Chhabra et al., 2011). Marketed formulation of cyclosporine (sandimmune neoral) in microemulsion form resulted in improved absorption of drug due to formation of mixed micelle after oral administration (Beveridge et al., 1981). Gorain et al. (2013) prepared Olmesartan medoxomil nanoemulsion to overcome low solubility of the drug apart from its conversion to less poorly permeable-form olmesartan which decreases the oral bioavailability of the drug. The pharmacokinetic study on rat showed 2.8 times increase in AUC and 3 times reduction in the dose (Gorain et al., 2013). Chhabra et al. (2011) prepared amlodipine besilate nanoemulsion and the concentration of the drug in heart and blood after 24 h of study was found higher for nanoemulsion formulation than drug suspension. Also the C_max_, AUC0→∞, and % relative bioavailability for the formulation were found to be 4.78, 2.2, and 475%, respectively. Jang D-J et al. enhanced the stability of amlodipine against photodegradation by formulating dry emulsion. Also, there self nanoemulsifying oily formulation (SNEOFs) was 2.9 times enhancement of bioavailability of the formulation of amlodipine (Jang D-J et al., 2006).

##### SNEDDS

SMEDDS and SNEDDS are the kinds of nanoemulsion as they in gastrointestinal milieu form nanoemulsion which is then up taken through lymphatic pathway (Beg et al., 2012). These systems consist of natural synthetic oil, surfactants, and co-surfactants. These LE are the isotropic mixtures of these excipients. Self-emulsifying systems seem more stable than nanoemulsion as they are not in direct contact with aqueous phase. Beg et al. prepared and found 143, 14, and 72% decrease in C_max_, AUC, and Ka, respectively while T_max_ increased by 1.8 times in the presence of cholestyramine when compared with formulation without cholestyramine. Cationic self nanoemulsifying oily formulation (CSNEOFs) result showed 27.4, 2.9, and 12.4% decrease in C_max_, AUC, and Ka, respectively, while T_max_ increased by 0.33 times in the presence of cholestyramine when compared with formulation without cholestyramine (Chhabra et al., 2011). Singh et al. (2013) prepared S-SNEDDS of carvedilol and estimated C_max_ and AUC as 134.2% and 85.2%, respectively in comparison to the drug suspension showing the potential of the formulation to enhance the oral bioavailability. Valsartan and lacidipine S-SNEDDS showed significant improvement in the dissolution profile. The bioavailability potential of valsartan S-SNEDDS was confirmed by significant reduction in the systolic blood pressure (systolic blood pressure) to 114.54 ± 1.84 mm of Hg after 0.5 h and 112.01 ± 2.27 mm of Hg in 2 h while the oral suspension reduced systolic blood pressure to 144.75 ± 1.85 mm of Hg after 0.5 h and 122.34 ± 2.26 mm of Hg in 2 h (Beg et al., 2012).

Ansari et al. prepared SMEDDS of felodipine for chronotherapeutic application. The rationale behind such approach was that hypertension shows circadian pattern, therefore the blood pressure does not remain similar for 24 h. It varies highest being in the morning and low during day time. SMEDDS using Lauroglycol FCC as oil and Cremophor EL/Transcutol P as surfactant/co-surfactant was prepared using dibutyl phthalate as plasticizer which gave a time lag of 5–7 h. So, pulsatile release of drug was achieved when there is maximum clinical manifestation of disease (Ansari et al., 2014).

##### Solid lipid nanoparticles

SLNs are composed of the excipients which are biocompatible and include solid lipid and surfactants/co-surfactants. Lipid excipients used are monoglycerides, diglycerides, and triglycerides of fatty acid with different chain length. More complex lipids are combination of these fatty acids with more imperfect crystal to accommodate more amount of drug into it. There are four different models of SLN which include SLN matrix, compound enriched shell, drug enriched core, and mixed type. Type of SLN formed depends upon the nature of drug and solid lipid used. The release of drug from SLN is biphasic, initial burst release followed by sustained release. The burst release can be minimized by decreasing manufacturing temperature and surfactant concentration. SLNs enhance the drug bioavailability by preventing first-pass metabolism as they undergo lymphatic uptake (Muller et al., 2000; Khan et al., 2015). Dudhipala et al. developed nisoldipine SLN. C_max_ and AUC_total_ of developed formulation is 12.55 ± 0.6 mg/mL and 96.15 ± 3.92 mg/mL/h while C_max_ and AUC_total_ of oral drug suspension is 7.53 ± 0.13 mg/mL and 44.13 ± 2.90 mg/mL/h. The oral bioavailability of the formulation was found to be 2.17 times higher than suspension. The researchers hypothesized that nanosized particle which adheres to the GI membrane increases residence time of SLN. The surfactants phosphatidylcholine and poloxamer enhance the permeability across the GI tract, apart from this the lipid used enhances the lymphatic uptake thereby circumventing the first-pass metabolism. Further pharmacodynamic study showed that SLN of nisoldipine significantly decreases the mean systolic blood pressure for a period of 36 h, revealing the sustained effect of formulation (Dudhipala & Veerabrahma, 2015). Zhang et al. showed enhancement in the C_max_ and AUC_0–t_ from 0.64 ± 0.15 μg/ml and 3.51 ± 0.87 μg·h/mL for candesartan suspension to 17.17 ± 2.40 μg/ml and 42.61 ± 7.53 μg·h/mL for SLN of candesartan. The AUC value indicates 12 times increase in oral bioavailability of the drug. T_max_ of SLN decreases to 0.42 ± 0.17 h from 2.75 ± 0.50 h. Such decrease indicates rapid drug absorption of the SLN than suspension thus making onset of action faster. The factor contributing to such an improved pharmacokinetic parameter was bioadhesiveness of SLN, intestinal permeation of SLN due to surfactant Tween 80, and nanosize range apart from lymphatic uptake (Zhang et al., 2012). Havanoor et al. prepared isradipine SLN and showed a marked decrease in the mean systolic blood pressure for a period of 12 h. Such studies show the potential of SLN for antihypertensive drugs as a long circulating nanocarrier which markedly improves the oral bioavailability and residence time of the drug (Havanoor et al., 2014).

##### Nanostructured lipid carrier

SLN has some limitation associated with it like expulsion of drug due to organization of solid lipid into more perfect crystal with time, which results in decrease in entrapment efficiency and loading capacity with time. This drawback associated with the SLN led to the development of NLC which is composed of liquid lipid apart from solid lipid. Liquid lipid is present within the solid lipid and does not undergo modification into stable structure; also solubility of drug in liquid lipid is higher than solid lipid, this results in enhancement of entrapment efficiency and loading capacity (Muller et al., 2000,2002; Selvamuthukumar & Velmurugan, 2012). NLC is generally of three types namely high imperfect matrix, multiple O/F/W type, and non-crystalline amorphous type based on the method of preparation. Ranpise et al. prepared NLC of poorly water-soluble drug lercanidipine hydrochloride having relative bioavailability of just 10%. There was significant reduction in blood pressure to 117.23 ± 1.61 mm of Hg after 8 h and 130.13 ± 1.97 mm of Hg after 24 h while plain drug administered to rats showed reduced blood pressure of 2.51 mm of Hg at 4 h and then rats developed hypertensive stage again. The reason behind such an effective result by NLC was its uptake by lymphatic route or peyer’s patches (Ranpise et al., 2014). In another study by Anuradha & Kumar showed better absorption of lacidipine NLC. AUC and C_max_ of lacidipine NLCs and lacidipine suspension was 8225 ng/ml/h, 813 ng/ml, and 2064.75 ng/ml/h, 571.77 ng/ml respectively. This high C_max_ and AUC value of NLC shows better drug absorption and increased relative bioavailability of around four times. These results show that NLC is a versatile nanocarrier and has the potential to incorporate antihypertensive with different physicochemical properties. NLC could be used as an alternative drug carrier in the antihypertensive drug delivery (Anuradha & Kumar, 2014).

##### Lipotomes

This is another lipid-based novel dual-functioning nanocarrier developed by ElKasabgy et al. for lacidipine, a poorly soluble drug. Lipotomes were prepared using lipid cetyl alcohol and surfactant Tween 80 by thin film hydration technique. Its dual function as claimed by the researchers is enhancement of drug solubility and bypassing first-pass metabolism of drug. Researcher compared enteric-coated lipotomes with enteric-coated lipid formulation without Tween 80 and marketed tablets. They found significant increase in the value of C_max_ of lipotomes (7.66 ± 3.52 ng/ml) than Tween 80 control preparation (3.62 ± 1.19 ng/ml), and marketed preparation (2.11 ± 0.81 ng/ml) showing efficacy of the lipotomes being absorbed efficiently. Also the relative bioavailability of lipotomes was 5.4 as compared to Tween control formula (relative bioavailability = 3.68). This result shows that the application of lipid excipient and surfactant Tween 80 individually plays vital role in enhancing clinical performance of the drug. The reason provided was that Tween 80 increases GI permeability, and lipid entrapped drug is circumvented by first-pass effect apart from its lymphatic uptake (ElKasabgy et al., 2014).

Thus, nanotechnology plays a big role in improving therapeutic efficacy of many therapeutics whether synthetic molecules or peptides. They enhance drug performance by either protecting their degradation or providing sustained release. Several challenges come in the way of nanoformulation such as scale-up, production cost, reproducibility, stability, and regulatory issues that still remain unaddressed (Desai et al., 2012).

### Nanoparticle-mediated gene therapy of hypertension

**Figure 3.**
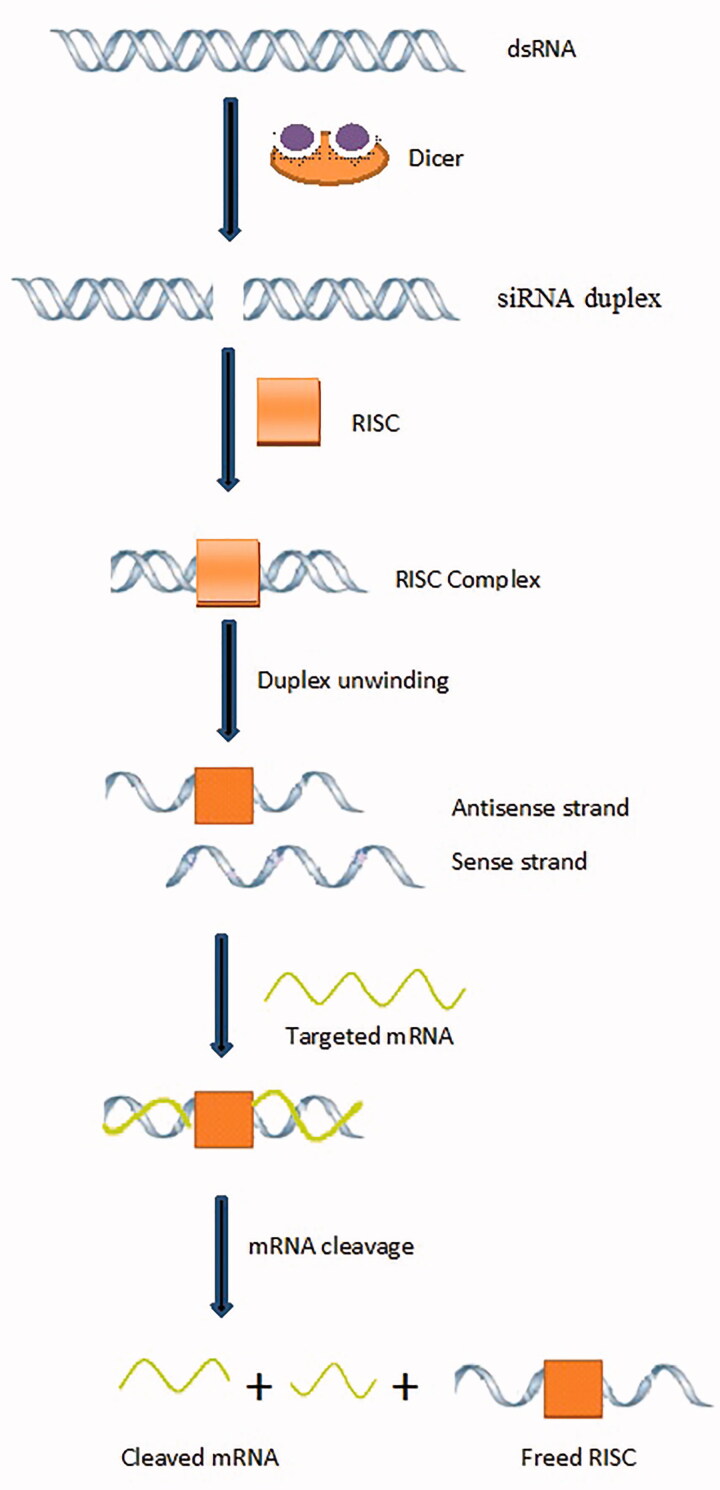
Mechanism of gene silencing.

Principle behind gene therapy is gene silencing. This refers to making target mRNA nonfunctional by its cleavage. The mechanism of gene silencing is shown in Figure 3. Small interfering RNA (SiRNA) is produced by RNAse III, and endonuclease also called Dicer. SiRNA duplex is incorporated into RISC (RNA-induced silencing complex), a nuclease resulting in RISC complex. SiRNA duplex undergo unwinding by RNA helicase resulting in antisense strand which remains with RISC (called activated RISC) while sense strand is degraded by exonuclease. This activated form of RISC binds with target mRNA and then RNAase activity is initiated by antisense strand of activated RISC. mRNA is cleaved into inactive fragments which become nonfunctional for protein synthesis. Thus gene silencing occurs, and receptor protein is not synthesized. Activated RISC becomes free to further destroy mRNA (Nolte et al., 2011; Koenig et al., 2013).

Gene therapy in hypertension refers to gene silencing of receptors which regulates the blood pressure. Small interfering RNA causes sequence-specific gene silencing thus receptor protein, which is the target of interest here is not synthesized. For example, AT1147siRNA was used by Vazquez et al. to silence AT1a, the subtype of angiotensin receptor, thus angiotensin II binding to this receptor is affected. siTRPC3 was another siRNA which decreased the expression of calcium-permeable transient receptor potential channel (TRPC). In another study, adeno-associated virus (AAV)-siRNA decreased the expression of AT1a receptor and mineralocorticoid receptor. siRNA effect was also seen in α1D-adrenergic receptor. The protein level of which was decreased by using siRNA (Nolte et al., 2011; Koenig et al., 2013). These studies show promising approach using siRNA for treatment of hypertension. The basic problem with siRNA is its rapid degradation upon administration. So, a delivery system is required to prevent degradation of siRNA by endo- and exonuclease present in the blood, serum, and cells. Lipoplex, a cationic liposome made of DOTAP (N-[1-(2,3-dioleoyloxy)]-N-N-N trimethyl ammonium propane), reduces the expression of β1-adrenoreceptor and controls the blood pressure for 12 days when given through intravenous route (Nolte et al., 2011).

## Conclusions and future perspectives

New generation antihypertensive drugs, new novel molecular targets and nanotechnology-based delivery system are currently in pivotal stage of preclinical trial and clinical trial and are showing positive results. Many novel molecular targets for antihypertensive are under exploratory phase and are being challenged with well-established already-existing antihypertensive therapy as far as their effectiveness is concerned. But there is still scope of improvement in therapy which can effectively control blood pressure. Nanotechnology is promising approach in resolving several constraints of antihypertensive. Targeted nanoparticle can effectively take antihypertensive to its site of action whether it is kidney, heart, or smooth muscle. Chronotherapeutics in conjunction with nanotechnology can effectively regulate the high blood pressure which can not only just modify the release pattern of drug but can also increase the bioavailability of drug. Gene silencing technology is innovative therapeutic tool which could definitely play a major role in future to treat hypertension. The challenges in gene delivery like cellular uptake and pharmacokinetics could be overcome by the use of suitable nanocarriers. However, oral drug delivery system of siRNA is still under its infancy for hypertension. But, several researches on different disease state using siRNA technology are developing very fast from preclinical to clinical trial level. Ultimately, success of the treatment depends upon the versatility of the nanoparticulate system which can entrap a wide variety of molecule including peptides and proteins and its targeting potential apart from its stability in external environment and in physiological condition.
